# Consumption of Micronutrient Powder, Syrup or Fortified Food Significantly Improves Zinc and Iron Status in Young Mexican Children: A Cluster Randomized Trial

**DOI:** 10.3390/nu14112231

**Published:** 2022-05-27

**Authors:** Armando García-Guerra, Juan A. Rivera, Lynnette M. Neufeld, Amado D. Quezada-Sánchez, Clara Dominguez Islas, Ana Cecilia Fernández-Gaxiola, Anabelle Bonvecchio Arenas

**Affiliations:** 1 Centro de Investigación en Nutrición y Salud, Instituto Nacional de Salud Pública (INSP), Universidad N-655, Colonia Santa María Ahuacatitlán, Cerrada los Pinos y Caminera, Cuernavaca 62100, Mexico; garciaf@insp.mx (A.G.-G.); anafdezg@hotmail.com (A.C.F.-G.); 2 Centro de Investigación en Salud Poblacional, Instituto Nacional de Salud Pública (INSP), Universidad N-655, Colonia Santa María Ahuacatitlán, Cerrada los Pinos y Caminera, Cuernavaca 62100, Mexico; jrivera@insp.mx; 3 Food and Agriculture Organization of the United Nations, 00153 Rome, Italy; lynnette.neufeld@fao.org; 4 Centro de Investigación en Evaluación y Encuestas, Instituto Nacional de Salud Pública (INSP), Universidad N-655, Colonia Santa María Ahuacatitlán, Cerrada los Pinos y Caminera, Cuernavaca 62100, Mexico; amado.quezada@insp.mx; 5 Fred Hutchinson Cancer Research Center, Vaccine and Infection Disease Division, 1100 Fairview Ave. N, Seattle, WA 98109, USA; cdomingu@fredhutch.org

**Keywords:** zinc, iron, fortified food, syrup, micronutrient powders

## Abstract

The objective of this study was to compare the effect of three micronutrient products on biomarkers of iron and zinc status of Mexican children 6–12 months of age. As part of research to improve the impact of a national program, 54 communities were randomly assigned to receive: (1) fortified food (FF), provided by the program at the time, or (2) micronutrient powders (MNP) or (3) syrup. Each product contained 10 mg each of zinc and iron, plus other micronutrients. Children consumed the product 6 days/week for four months. Primary outcomes were changes in serum zinc, ferritin, soluble transferrin receptor, hemoglobin concentrations, and their deficiencies. Zinc concentration increased significantly from baseline to follow-up in all groups, with the largest change in the syrup group (geometric mean difference: +4.4 µmol/L; 95%CI: 3.2, 5.5), followed by MNP (+2.9 µmol/L; 95%CI: 2.1, 3.6) and FF (+0.9 µmol/L; 95%CI: 0.3, 1.6). There was a significant increase in hemoglobin concentration (+5.5 g/L; 2.5, 8.4) and a significant reduction in anemia prevalence (44.2% to 26.8%, *p* < 0.01) only in the MNP group. Compliance differed significantly among groups (MNP vs. FF, *p* = 0.04; MNP vs. syrup, *p* = 0.04), but may not fully explain the greater improvement in zinc and iron status in the syrup and MNP groups. The food matrix may influence nutrient utilization from supplements.

## 1. Introduction

Worldwide, 30% of children under five years of age have zinc deficiency, and 44% have anemia [1,2], of which approximately 42% is due to iron deficiency [2]. Reducing iron deficiency in children may improve several functional outcomes, including mental and motor development [3]. Zinc deficiency affects children’s physical growth and may increase the risk and severity of a variety of infections, particularly causing diarrhea and pneumonia [1].

In Mexico, 10.8% of children 1–4 years of age had a low serum zinc concentration (<65 µg/dL) and 20.4% were anemic in 2018 [4]. Iron deficiency was the major cause of anemia in this population group, with 16% of children having iron deficiency anemia [5]. The *Prospera Programa de Inclusión Social* (formerly *Oportunidades* Human Development Program and initially *Progresa*) was a large-scale social protection program that benefited approximately 6 million Mexican families from 1997 to 2018. Beneficiary households received financial, medical, and nutritional support conditional on utilization of preventative health services, and attending educational programs on health, hygiene, and nutrition, among other benefits. Eligibility was established by residence in a community serviced by the program and living in poverty, defined by a series of criteria that reflected insufficient assets and income to invest in the development of the household members [6]. At the time of this study, all children in beneficiary households received a fortified complementary food (*Nutrisano*) from 6 to 23 months of age, and 2 to 4 years if underweight. Previously reported benefits of the *Oportunidades* program include moderate growth, anemia, and development of children [7,8,9,10]. Evidence suggested that *Nutrisano* was consumed at a low frequency (<4 times/week) or at lower levels (~20 g/day) than the daily recommendation of 44 g, mainly due to sharing with other family members [11].

It has been suggested that home fortification through the provision of micronutrient powder (MNP) directly into a child’s food may be a more cost-efficient intervention to improve the quality of home-based methods to reduce micronutrient deficiencies [12]. This may be particularly relevant in a context like Mexico, where acute malnutrition is not a public health concern [13]. The efficacy of MNPs to preventing and control anemia has been demonstrated in many settings, and program effectiveness has been demonstrated in a number of countries [14]. Micronutrient syrups have also been used extensively, but compliance with utilization tends to be low.

The objective of this study was to determine the relative efficacy of alternative carriers of micronutrients (fortified food, MNP, or syrup), in improving biochemical indicators of micronutrient status (serum ferritin, soluble transferrin receptor, and serum zinc concentrations) among children 6–12 months of age in urban areas of southern Mexico after four months of daily consumption. This study was conducted within a large trial with longer supplementation and follow-up period, the results of which were published previously [15]. 

## 2. Materials and Methods

### 2.1. Setting and Design

A detailed description of the design, study population, intervention, information collection, and primary outcomes has been published previously [15]. Briefly, the study was a cluster-randomized trial [15], where urban communities (*n* = 54) were randomly allocated within each design block to one of the three interventions groups: (1) fortified food (FF), *Nutrisano*, the multiple micronutrients fortified food provided through *Prospera*; (2) micronutrients powder (MNP); or (3) micronutrient syrup (syrup) (Table 1). The trial (Clinical Trial Registry NCT00531674) could not be blinded due to the different presentation of the supplements. 

Study participants: Eligible participants included all children six to 12 months of age at the time of recruitment in participating communities. Physicians and nurses at the health care centers identified beneficiaries of the *Prospera* program for potential inclusion and organized informational meetings to facilitate discussion. Children with severe anemia (hemoglobin < 90 g/L) were excluded; these children were referred for medical follow-up. This biomarker sub-study includes data from baseline and after four months of supplementation. All children whose caregivers provided consent for the venous blood draw were included in this sub-study. 

Informed consent: All caregivers received complete details of the objectives, procedures, potential risks, and benefits of the study. The consent declaration was read to the caregivers who expressed interest in the study, and if agreed, they signed or provided a fingerprint on the consent form to confirm voluntary consent for the full study, and then separately for this sub-study’s blood draws. 

Interventions: Prior to the study, all families received the fortified food as per are criteria mentioned as part of the *Prospera* benefits [6]. Once the study commenced, regular educational messages continued through the *Prospera* program, but the product (FF, MNP, syrup) randomly assigned by the community was delivered daily (6 days/week) by trained field workers directly to the home and consumption was observed and recorded. The timing of home visits was agreed upon with the caregiver to minimize interference with regular meals. 

This trial was designed to specifically address the question of which type of supplement to provide for decision-making in the program. Although willing to consider alternative supplements, the program implementers were not considering the possibility of eliminating the supplement and therefore did not agree to the inclusion of a placebo control group. 

Each product contained 10 mg equivalent of elemental iron and zinc, plus equivalent amounts of vitamins A, E, C, B2, B12, and folic acid (Table 1). Because the FF was formulated with the whole dry milk powder, sugar, and maltodextrin, children in sites randomized to the FF group also received supplementary energy, macronutrients, calcium, and several other nutrients naturally occurring in the food ingredients.

The FF was produced by *Liconsa* Corporation (*S.A. de C.V.*, *Querétaro, Qro.*, *México*) and distributed in 264 g bags. The recommended daily dose was 44 g of dry product per day to be mixed with a small amount of water immediately prior to consumption. The syrup (5 mL/day, distributed in 60-mL bottles) was developed specifically for this project and donated by Laboratories Zerboni (Mexico City, Mexico) using premix donated by DSM Nutritional Products Mexico (Mexico City, Mexico). MNP was produced specifically for this study and purchased from Ped-Med Ltd. (Toronto, Canada). The 1 g/day dose was provided in individual daily sachets to be mixed with a small portion of the child’s semi-solid food immediately prior to consumption. 

### 2.2. Data Collection and Descriptions

Detailed household demographic and socioeconomic status (SES) information and the child’s morbidity history were collected at baseline. Specific questions identified household materials, availability and use of health services, family assets, maternal and paternal education level and occupation, and participation in any other social programs. 

At baseline and after 4 months of supplementation, weight and recumbent length were measured by trained and standardized anthropometrics following standard measurement protocols [16]. Weights were measured to the closest 0.1 kg using Tanita electronic scales (Tanita Corp., Arlington Heights, IL, USA). Infants or children not yet able to stand were measured in their mother’s arms using the tare function. Recumbent length was measured in duplicate to the nearest 0.1 cm using a portable infantometer (Short Productions, Olney, MD, USA).

Venous blood samples (7 mL) were collected at baseline and followed up by trained phlebotomists and stored using trace element-free collection tubes (Vacutainer, Becton Dickenson, Franklin Lakes, NJ, USA). The samples were collected in the morning and the mother’s estimate of time since the child’s last meal was recorded; fasting samples were not feasible due to field logistics. Blood samples were stored on ice for 20–30 min before centrifugation at 2000 rpm at room temperature in the field clinic. Serum was immediately transferred to trace element-free microtubes and quickly frozen in liquid nitrogen. Samples were then transported on liquid nitrogen to the nutrition laboratory of INSP where they were stored at −70 °C until biochemical analysis. 

Hemoglobin concentration from capillary blood samples (HemoCue B-Heoglobin, AB) was available at multiple time points (baseline, 2, 4, 10 months supplementation, and at age 24 months) [15]. In this analysis, we used the baseline measurement and then at 4 months after the start of supplementation.

Laboratory analyses to determine micronutrient concentrations were done at the INSP nutrition laboratory by trained laboratory technologists. Serum ferritin, soluble transferrin receptor (sTfR), and C-reactive protein (CRP, mg/L) were determined by the nephelometry method (Behring Nephelometer B-N 100, Marburg, Germany). Serum zinc was determined by inductively coupled plasma atomic emission spectroscopy (ICP-AES, Varian, Vista Pro CCD-Simultaneus, Mulgrave, Victoria, Austria). 

### 2.3. Data Management, Statistical Analysis, and Power

#### 2.3.1. Data Management

Outcomes of interest for this analysis are serum concentrations of zinc (µmol/L), ferritin (µg/L), soluble transferrin receptor (mg/L), and hemoglobin concentration (g/L). Zinc deficiency was defined as serum zinc below 9.9 µmol/L [17]. Low iron stores were defined as ferritin below 12 µg/L and tissue iron deficiency as sTfR above 6 mg/L [18,19,20]. Anemia was defined as a hemoglobin concentration <110 g/L and adjusted for community altitude above sea level [18,20].

An index of socioeconomic status was constructed (using the full sample) by applying principal components analysis on a set of variables related to household materials (floor, walls, and ceiling), services (water source, electricity, and sanitary facilities), and durable goods (e.g., automobile, motorcycle, television, stereo, blender, refrigerator, etc.). The first of the resulting principal components were selected. Height-for-age, length-for-age, and weight-for-length were compared to the 2006 WHO Child Growth Standards. Stunting Z-score was defined as a length-for-age less than −2 standard deviation (SD) below the reference median, and overweight or obesity Z-score as BMI for age greater than 2 SD above the reference median [21]. No children in this study were classified as wasted (weight-for-length < −2 SD below the reference median).

Supplement compliance over the four months of intervention was calculated by dividing the sum of partial and full doses consumed by the total doses offered (% of doses consumed). 

#### 2.3.2. Statistical Analysis 

We compared study groups in terms of their baseline characteristics such as age, sex, a socioeconomic index, nutritional status, and CRP. We calculated means and standard deviations for continuous variables and percentages for categorical variables. For compliance to supplementation and baseline CRP, we calculated median and interquartile range (P25, P75). 

We compared subjects that were included in the sub-study with those that were not included through t-tests for means and z-tests for proportions. Median tests based on contingency tables and adjusted for clustered data within communities were performed for compliance to supplementation by the study group.

Geometric means (or prevalence for binary outcomes) were estimated at baseline and follow-up, as well as their changes. Given the study objective of selecting among three alternative micronutrient products, and the absence of a placebo control group, pair-wise contrasts of changes in geometric means among study groups were interpreted as differential effects. We also tested for changes from baseline to follow-up in each study group. 

Estimates were obtained from multiple linear regressions using the natural log transformation for continuous outcome variables and logistic regressions for binary outcome variables. The linear predictor in all models included an indicator variable of the 4 months period, indicator variables of the study group (FF as reference category), and their interactions with the study period. Adjustment covariates included those characteristics that were identified to be unbalanced between groups at baseline. Standard errors for all models were adjusted for data clustering within communities. Geometric means (or prevalence for binary outcomes), their changes, and their contrasts between study groups were obtained from predictive margins and their standard errors were calculated with the Delta method [22,23]. Predictive margins allow the comparison of categories of a variable of interest while holding constant the distribution of the covariates. This is done by leaving the covariates in their observed values and replacing the variable of interest with one of its values in the linear predictor, then the function to obtain the desired estimate is applied to each observation and these values are averaged over the whole sample. This process is repeated for each of the values of the variable of interest. This approach is particularly useful for models in which the desired statistic is a non-linear transformation of the linear predictor (e.g., applying the inverse logit function to the linear predictor in logistic regression to obtain adjusted probabilities). 

Study design blocking indicator variables were included along with the other predictors in all models. Data management, processing, and statistical analyses were done using Stata 17 (StataCorp. 2021. Stata Statistical Software: Release 17. College Station, TX, USA: StataCorp LLC).

#### 2.3.3. Statistical Power

The sample size for the main trial was calculated based on the primary endpoints (child growth) [15]. Assuming 70 observations in one study group and 100 in another study group, a standardized effect size of 0.4, an intra-cluster correlation of 0.06 (or a design effect of 1.3), and a correlation of 0.6 between baseline and 4 mo follow-up outcome measurements, the study has a power of at least 71% for detecting a difference of mean changes between any two groups. 

### 2.4. Ethical Considerations

The study was designed and implemented by researchers at the *Centro de Investigación en Nutrición y Salud* (CINyS) from *Instituto Nacional de Salud Pública* (INSP) in *Cuernavaca, Morelos, Mexico,* and ethical approval was obtained from the Research, Ethical and Biosecurity Commissions (CI-213) at INSP.

## 3. Results

Of the 988 children who eligible to participate in the full trial, 928 started the trial (FF: 288, MNP: 352, and syrup: 348). This study includes 283 children with a venous blood sample at baseline and after 4 months (FF: 73; MNP: 105; syrup: 105). An analysis comparing baseline characteristics and supplement compliance between included and excluded children is available as supplementary material (Table S1). 

The average age of participants in the sub-sample was 8.4 ± 2.5 (±SD) months at baseline and the prevalence of stunting was 17.3% (Table 2). Households from the syrup group had a lower mean socioeconomic index than those in the FF and MNP groups. Median baseline CRP concentration was lower in the MNP group than in the FF group. 

Median and interquartile range (P25, P75) of supplement compliance was 87% (78.4%, 94.6%) in the MNP group, 78.8% (57.5%, 89.6%) in the syrup group and 73.5% (48.8%, 90.6%) in the FF group. Median compliance was significantly higher in the MNP than the FF (*p* = 0.04) and the Syrup group (*p* = 0.04). 

We completed the follow-up (4 months) biochemical analyses on 94–96% of participants with baseline assessments. Results of biochemical analyses (Table 3 and Table 4) include data from participants with sufficient blood to analyze both baseline and four-month follow-up for each indicator (*n*: Syrup = 100; FF = 73; MNP = 104).

### Differential Effects of Supplementation

There was a significant and positive change in serum zinc concentrations from baseline to four months of follow-up among children in each of the three intervention groups (Table 3). All pairwise comparisons were statistically significant (i.e., none of the corresponding confidence intervals included zero), with the largest change in the syrup group, followed by MNP and FF groups. Geometric mean ferritin concentration at follow-up was lower in the FF group than in both MNP and syrup groups. Compared to FF, geometric mean hemoglobin concentration at follow-up and its change were significantly higher in the MNP group.

The prevalence of zinc deficiency decreased in all three groups (Table 4) with a larger decrease in the Syrup group compared to the FF group. The prevalence of low iron stores (defined as serum ferritin <12 µg/L) at follow-up was higher in the FF group than in the syrup and MNP groups. No differential effects of the supplement group were found for the prevalence of tissue iron deficiency. The prevalence of anemia decreased significantly in the MNP group only. The change in anemia prevalence was significantly different between the FF and MNP groups (−17.4 vs. +4.1, *p* < 0.05). These results did not differ with no adjustment for unbalanced characteristics between groups at baseline (Supplementary Materials: Tables S2 and S3). 

## 4. Discussion

In this cluster randomized trial, consumption of any one of the three supplements for four months significantly improved zinc and iron status in the subsample of children with biochemical data. Syrup and MNP were more efficacious than FF to reduce zinc and iron deficiency. Only MNP showed a statistically significant decrease in anemia prevalence, although the level of precision of estimates did not allow conclusive results for the other study groups. These differences reflect the higher level of compliance in MNP. Furthermore, the matrix in which supplements are provided may have implications for their absorption or utilization, as has been reported previously [24,25]. 

One of the unique features of the products used is that they contained 10 mg each of both iron and zinc. The zinc content of the FF as originally designed for the program was included in response to the high documented prevalence of zinc deficiency in Mexico at the time [4]. The other products used in this study were developed to match that nutrient composition. Trials with lower doses of zinc have found mixed effects on zinc status, with many reporting no change [26,27]. We found only one other trial using 10 mg of zinc in the MNPs, in Pakistan [24]. Our reported magnitude of change in plasma zinc concentration is substantially larger than that study. This may be due to the high compliance achieved with our community-based efficacy trial; compliance was not quantified in the trial in Pakistan. 

Our comparison of three products also allowed us to observe differential effects in impact on zinc concentrations being highest in children who consumed syrup, followed by MNP, and lowest in fortified food (Table 3). As noted, this may imply differences in absorption or nutrient utilization depending on the supplement type. Because there was no placebo group, we cannot rule out the possibility that the observed changes over time in serum zinc concentrations among children could be due to the natural increase associated with age or other factors. That would not, however, explain the differential impact among products. Population-level data for very young children are not available for comparison, but the differences that have been reported in older age groups indicate that the 6.1 μg/dL (0.93 μmol/L) mean increase observed among children consuming the FF for example, is more likely to occur naturally over several years rather than a short four-month period [17]. 

The positive impact of MNP or syrup consumption on iron status biomarkers is consistent with the observations among children in previous studies [14,28]. The lack of change from baseline to follow-up in the FF group may be due to interference in the absorption of iron from the food matrix, for example, due to calcium and casein naturally occurring in milk [29]. The competition between zinc and iron, which has been cited as a concern [16,30], seems unlikely in this case given the results for MNP and syrup. Other studies have also confirmed that competition for absorption between iron and zinc is unlikely, except at very high ratios [31].

Some recent studies have documented adverse effects of MNPs on morbidity, particularly diarrhea [14]. Preliminary analysis of reported morbidity from this trial found no evidence of differential incidence by the group [32]. It is possible that for this population in Mexico, with adequate access to health care (a core benefit of *Prospera*) and better nutrition and health profile generally, the iron provided in MNPs does not increase the risk of morbidity. Confirmation of this, however, requires additional study. 

The ultimate goal of this study was to identify whether alternative supplements that may have lower cost and/ or more favorable consumption patterns without compromising benefits may result in cost-efficiencies for the program [15]. Despite daily delivery by the highly-trained field staff, the total number of doses consumed by the MNP group was higher in the 4 months than either of the other supplements. Based on our qualitative studies and the team’s observation, MNP was highly accepted by caregivers, perhaps due to the flexibility in its use (e.g., adding to diverse and small quantities of food) and because it does not modify the taste, acceptance can be better than syrup [33]. 

Some limitations of this study should be acknowledged. The fact that children in the syrup group reportedly lived in conditions with lower socioeconomic conditions than children in the other two groups may have put them at greater nutritional or health risk, thus with greater potential to benefit from the products. Although, in the data analyses the adjustment was made for socioeconomic conditions. The results without adjustment are in the supplementary material, which shows results similar to the results with this adjustment. The greater effect of syrup, however, was only observed for serum zinc concentrations, which may have already been anticipated given previous research [34,35]. Due to the randomization scheme of the main trial and the similar inclusion criteria (informed consent for this sub-study), we do not feel that the “convenience” of this sub-study’s sampling has led to any important bias in the findings. The statistically significant difference in the age of children in this sub-study compared to children in the full study (0.4 months) is not expected to be important when evaluating whether the outcomes observed in this sub-group would be similar among the larger set of children. However, the magnitude of the changes observed might differ by age as the risk of deficiency will vary. 

This study was part of a series to inform changes to the nutrition component of the *Prospera* conditional cash transfer program and as such did not include a placebo control. Specifically, the studies were designed to address the challenge of low regular consumption of the FF by the target children (6 to 24 months of age) and to explore alternatives that might provide at least similar benefits while improving targeting and regular consumption and reducing program costs. In this population with a high prevalence of iron deficiency anemia (≥40%) [18], all three supplements improved the nutritional status, with slightly greater improvements in the MNP and syrup groups. The ultimate utility of this study for policy change in the program is interpreted in light of many other outcomes and have been published previously [36]. 

## Figures and Tables

**Table 1:** Nutritional content of the supplements ^1^.

	*Nutrisano* (Fortified Food)	Micronutrient Powder	Syrup
Quantity to be consumed	(44 g)	(1.0 g)	(5 mL)
Energy, kcal	194	-	-
Protein, g	5.8	-	-
Carbohydrates, g	27.9	-	-
Lipid, g	6.6	-	-
Sodium, mg	24.5	-	-
Iron, mg ^2^	10.0	10.0	10.0
Zinc, mg ^3^	10.0	10.0	10.0
Vitamin A, µg ER	400.0	400.0	400.0
Vitamin E, µg ET	6.0	6.0	6.0
Vitamin C, mg	50.0	50.0	50.0
Vitamin B_2_, mg	0.8	0.8	0.8
Vitamin B_12_, µg	0.7	0.7	0.7
Folic acid, µg	50.0	50.0	50.0

^1^ Micronutrients added to all three products are expected to supply 100% of these micronutrients daily for this age group. *Nutrisano* (fortified food) was formulated to supply 20% of energy. ^2^ Iron in the fortified food and the syrup was in the form of ferrous gluconate; in the micronutrient powder, ferrous fumarate was used. ^3^ Zinc gluconate was used in all three products.

**Table 2:** Baseline characteristics of the sub-sample of participants with venous blood samples at baseline and after 4 months of supplementation with one of three micronutrient products.

	Fortified Food	Syrup	MNP ^1^	Total
	*n* = 73	*n* = 105	*n* = 105	*n* = 283
General characteristics				
Age, months	8.2 ± 2.5	8.5 ± 2.6	8.4 ± 2.4	8.4 ± 2.5
Sex, % males	47.9	47.1	55.2	50.2
Socioeconomic Index ^#^	0.2 ± 1.0	−0.2 ± 1.0	0.1 ± 1.0	0.0 ± 1.0
Anthropometric measurements				
Length, cm	67.3 ± 4.3	67.5 ± 4.5	67.8 ± 4.2	67.6 ± 4.3
Weight, kg	7.9 ± 1.2	7.8 ± 1.3	8.1 ± 1.2	7.9 ± 1.2
Nutritional Status				
Length for age, Z	−1.0 ± 1.0	−1.1 ± 1.0	−1.0 ± 1.0	−1.0 ± 1.0
Stunting ^2^, %	17.8	14.4	20.0	17.3
Weight for age, Z	−0.4 ± 1.0	−0.6 ± 1.0	−0.4 ± 1.0	−0.5 ± 1.0
Weight for length, Z	0.3 ± 0.9	0.1 ± 1.0	0.3 ± 0.9	0.3 ± 0.9
BMI for age, Z	0.3 ± 0.9	0.1 ± 1.0	0.3 ± 0.9	0.2 ± 0.9
Overweight or obesity ^3^, %	2.7	1.0	1.9	
CRP, mg/L				
Median	1.7	1.1	0.8	1.0
[p25, p75]	[0.4, 8]	[0.3, 4.2]	[0.2, 3.4]	[0.3, 4.2]

Estimates are mean ± standard deviation or prevalence, unless otherwise stated. ^1^ MNP: micronutrient powder. ^2^ Stunting defined as length for age Z score below-2. ^3^ Overweight or obesity defined as BMI for age Z score above 2. ^#^ Socioeconomic status index obtained from a principal component analysis of household characteristics and possessions of durable goods, with the first component explaining 17% of the total variance.

**Table 3:** Geometric mean concentration of serum zinc, ferritin, soluble transferrin receptor, and hemoglobin at baseline and follow up samples measured at 4 months, by study group.

	FF	Syrup	MNP ^1^	Syrup vs. FF	MNP vs. FF	MNP vs. Syrup
Zinc, µmol/L						
	*n* = 73	*n* = 99	*n* = 104			
Baseline	11.3 (10.9, 11.8)	10.8 (10.5, 11.2)	10.9 (10.4, 11.3)	−0.5 (−1.1, 0.1)	−0.5 (−1.1, 0.2)	0.0 (−0.6, 0.6)
4 months	12.3 (11.9, 12.7)	15.2 (14.3, 16.2)	13.7 (13.2, 14.2)	2.9 (1.9, 4.0)	1.5 (0.8, 2.1)	−1.5 (−2.6, −0.4)
Change	0.9 (0.3, 1.6)	4.4 (3.2, 5.5)	2.9 (2.1, 3.6)	3.4 (2.1, 4.7)	1.9 (0.9, 2.9)	−1.5 (−2.9, −0.1)
Ferritin, µg/L						
	*n* = 70	*n* = 98	*n* = 99			
Baseline	18.7 (12.7, 24.7)	26.5 (20.0, 32.9)	22.3 (16.7, 27.8)	7.8 (−1.4, 16.9)	3.6 (−4.7, 11.8)	−4.2 (−12.9, 4.4)
4 months	14.5 (10.5, 18.6)	24.7 (19.8, 29.6)	25.7 (20.2, 31.2)	10.2 (3.5, 16.8)	11.2 (4.3, 18.1)	1.0 (−6.5, 8.6)
Change	−4.2 (−11.6, 3.3)	−1.8 (−7.6, 4.0)	3.5 (−5.6, 12.6)	2.4 (−7.1, 11.8)	7.6 (−4.1, 19.4)	5.3 (−5.5, 16.0)
sTfR, mg/L						
	*n* = 70	*n* = 99	*n* = 99			
Baseline	4.14 (3.44, 4.83)	4.73 (4.27, 5.20)	4.52 (4.17, 4.86)	0.60 (−0.25, 1.45)	0.38 (−0.42, 1.19)	−0.22 (−0.82, 0.39)
4 months	4.87 (4.35, 5.40)	4.36 (4.15, 4.57)	4.49 (4.20, 4.77)	−0.51 (−1.09, 0.06)	−0.39 (−0.99, 0.21)	0.13 (−0.23, 0.49)
Change	0.74 (−0.23, 1.71)	−0.38 (−0.88, 0.12)	−0.03 (−0.53, 0.47)	−1.11 (−2.21, −0.02)	−0.77 (−1.86, 0.32)	0.34 (−0.36, 1.05)
Hemoglobin, g/L						
	*n* = 73	*n* = 100	*n* = 103			
Baseline	110.6 (108.0, 113.2)	109.2 (105.6, 112.7)	110.2 (107.7, 112.6)	−1.4 (−5.9, 3.0)	−0.4 (−4.0, 3.2)	1.0 (−3.3, 5.4)
4 months	109.3 (107.4, 111.3)	113.6 (110.6, 116.6)	115.6 (114.0, 117.3)	4.3 (0.7, 7.8)	6.3 (3.5, 9.1)	2.1 (−1.5, 5.6)
Change	−1.3 (−4.1, 1.6)	4.4 (−1.4, 10.3)	5.5 (2.5, 8.4)	5.7 (−0.8, 12.2)	6.7 (2.7, 10.8)	1.0 (−5.5, 7.6)

95% confidence intervals are shown in parentheses. Estimates were obtained from multiple linear regressions using the natural log transformation for outcome variables, the linear predictor included an indicator variable of the 4 months period, indicator variables of the study group (FF as reference category), and their interactions with the study period. Study blocking indicator variables were included along with the other predictors. Adjustment covariates included the log of baseline CRP and a socioeconomic status index. Standard errors in the model were adjusted for data dependencies within communities. Covariate-adjusted geometric means, their changes, and their comparisons were performed through predictive margins, and their standard errors were calculated with the Delta method. ^1^ MNP: Micronutrient powder.

**Table 4:** Prevalence of zinc deficiency, low iron stores, tissue iron deficiency, and anemia, at baseline and 4 mo follow up, by study group.

	FF	Syrup	MNP ^1^	Syrup vs. FF	MNP vs. FF	MNP vs. Syrup
Zinc deficiency (Zn < 9.9 µmol/L), %						
	*n* = 73	*n* = 99	*n* = 104			
Baseline	22.9 (14.2, 31.6)	39.2 (31.0, 47.5)	32.1 (24.0, 40.3)	16.4 (3.9, 28.9)	9.3 (−3.0, 21.5)	−7.1 (−19.4, 5.2)
4 months	10.8 (4.0, 17.5)	3.0 (0.1, 5.9)	8.6 (4.7, 12.5)	−7.8 (−15.1, −0.4)	−2.2 (−10.2, 5.9)	5.6 (0.8, 10.4)
Change	−12.1 (−22.6, −1.6)	−36.2 (−45.8, −26.6)	−23.5 (−34.3, −12.8)	−24.1 (−38.8, −9.4)	−11.4 (−26.4, 3.5)	12.7 (−2.2, 27.6)
Low iron stores (ferritin < 12 µg/L), %						
	*n* = 70	*n* = 98	*n* = 99			
Baseline	33.8 (21.2, 46.3)	27.9 (17.4, 38.4)	25.7 (18.3, 33.1)	−5.9 (−22.6, 10.8)	−8.1 (−22.9, 6.7)	−2.2 (−15.2, 10.8)
4 months	39.8 (26.5, 53.0)	15.2 (9.2, 21.2)	14.8 (7.1, 22.4)	−24.6 (−39.7, −9.5)	−25.0 (−40.2, −9.8)	−0.4 (−10.5, 9.7)
Change	6.0 (−11.4, 23.3)	−12.7 (−22.2, −3.2)	−10.9 (−24.1, 2.3)	−18.7 (−38.5, 1.1)	−16.9 (−38.6, 4.8)	1.8 (−14.4, 18.0)
Tissue iron deficiency (sTfR > 6 mg/L), %						
	*n* = 70	*n* = 99	*n* = 99			
Baseline	16.3 (3.3, 29.2)	19.6 (9.4, 29.8)	18.1 (9.2, 27.1)	3.3 (−13.6, 20.3)	1.9 (−14.9, 18.6)	−1.5 (−15.0, 12.1)
4 months	16.3 (7.4, 25.1)	7.8 (2.5, 13.1)	6.5 (0.9, 12.1)	−8.5 (−18.7, 1.8)	−9.8 (−20.5, 1.0)	−1.3 (−9.2, 6.6)
Change	0.0 (−10.8, 10.8)	−11.8 (−24.4, 0.9)	−11.6 (−23.1, −0.2)	−11.8 (−28.5, 4.9)	−11.6 (−27.4, 4.1)	0.2 (−16.8, 17.1)
Anemia (Hb < 110 g/L), %						
	*n* = 73	*n* = 100	*n* = 103			
Baseline	49.3 (42.9, 55.7)	48.4 (37.5, 59.4)	44.2 (33.2, 55.2)	−0.9 (−13.9, 12.2)	−5.1 (−18.5, 8.3)	−4.2 (−20.0, 11.5)
4 months	53.4 (46.7, 60.1)	35.5 (24.5, 46.5)	26.8 (21.6, 32.0)	−17.9 (−31.1, −4.7)	−26.6 (−35.4, −17.8)	−8.7 (−21.3, 3.9)
Change	4.1 (−4.1, 12.2)	−12.9 (−31.8, 5.9)	−17.4 (−29.6, −5.2)	−17.0 (−37.5, 3.5)	−21.5 (−36.2, −6.8)	−4.5 (−26.9, 17.9)

95% confidence intervals are shown in parentheses. Estimates were obtained from logistic regressions, the linear predictor included an indicator variable of the 4 months period, indicator variables of the study group (FF as reference category), and their interactions with the study period. Study blocking indicator variables were included along with the other predictors. Adjustment covariates included the log of baseline CRP and a socioeconomic status index. Standard errors in the model were adjusted for data dependencies within communities. Covariate-adjusted prevalence, their changes, and their comparisons were performed through predictive margins, and their standard errors were calculated using the Delta method. ^1^ MNP: Micronutrient powder.

## Data Availability

The data presented in this study are available on request from the corresponding author.

## Supplementary Materials

The following supporting information can be downloaded at: https://www.mdpi.com/article/10.3390/nu14112231/s1, Table S1: Baseline characteristics of children from the full trial according to participation status in the study with venous blood collection; Table S2: Geometric mean concentrations of serum zinc, ferritin, soluble transferrin receptor (sTfR) and hemoglobin in baseline and follow up samples measured at 4 months without covariate adjustment, by study group; Table S3: Prevalence of zinc deficiency, low iron stores, tissue iron deficiency and anemia in baseline and follow up samples measured at 4 months without covariate adjustment, by study group.
