# Supplementary material for: Consumption of Micronutrient Powder, Syrup or Fortified Food Significantly Improves Zinc and Iron Status in Young Mexican Children: A Cluster Randomized Trial
Source: Nutrients. 2022 May 27;14(11):2231. doi: 10.3390/nu14112231 (PMC9183015; doi:10.3390/nu14112231)
Supplement: Supplementary file 1 [file nutrients-14-02231-s001.zip › nutrients-1639868-supplementary.pdf]

Supplementary Table S1. Baseline characteristics of children from the full trial according to participation status in the study with venous blood collection

	Participants in sub-study	Non-participants in sub-study
	n=283	n=648
**General characteristics**		
Age, months	8.4 ± 2.5	8.0* ± 2.6
Sex, % males	50.4	47.8
Socioeconomic Index#	0.0 ± 1.0	0.0 ± 1.0
**Anthropometric measurements**		
Length, cm	67.6 ± 4.3	66.9* ± 4.2
Weight, kg	7.9 ± 1.2	7.9 ± 1.2
**Nutritional Status**		
Length for age, Z	-1.1 ± 1.0	-1.1 ± 1.1
Stunting,[1] %	17.4	18.5
Weight for age, Z	-0.5 ± 1.0	-0.4 ± 1.0
Weight for length, Z	0.3 ± 0.9	0.4 ± 1.0
BMI for age, Z	0.2 ± 0.9	0.3 ± 1.0
Overweight or obesity,[2] %	1.8	3.9

*p<0.05 between groups from t-tests for means.
Socioeconomic status index obtained from a principal component analysis of household characteristics and possessions of durable goods, with the first component explaining 17% of the total variance.
1. Stunting defined as length for age Z score below -2.
2. Overweight or obesity defined as BMI for age Z score above 2.
Participants included in this sub-study were slightly older and had higher average length than those that were not included. There were no other significant differences in base-line characteristics according to participation status. Regarding supplement compliance defined as the percentage of dose actually consumed over the total days observation, the median (P25, P75) compliance was 82.1% (64.3%, 93.0%) for participants and 81.4% (57%, 93.3%) for non-participants.

Supplementary Table S2. Geometric mean concentrations of serum zinc, ferritin, soluble transferrin receptor (sTfR) and hemoglobin in baseline and follow up samples measured at 4 months without covariate adjustment, by study group

	Fortified Food	Syrup	MNP[1]	Syrup vs FF	MNP vs FF	MNP vs Syrup
**Zinc, µmol/L**						
	n=73	n=104	n=105			
Baseline	11.3 (10.8, 11.8)	10.8 (10.4, 11.1)	10.9 (10.4, 11.3)	-0.5 (-1.2, 0.1)	-0.4 (-1.1, 0.2)	0.1 (-0.5, 0.7)
4 months	12.2 (11.9, 12.6)	15.2 (14.3, 16.2)	13.8 (13.3, 14.3)	3.0 (2.0, 4.0)	1.5 (1.0, 2.1)	-1.4 (-2.5, -0.3)
Change	0.9 (0.3, 1.6)	4.5 (3.2, 5.7)	2.9 (2.2, 3.7)	3.5 (2.2, 4.9)	2.0 (1.0, 3.0)	-1.6 (-3.0, -0.1)
**Ferritin, µg/L**						
	n=70	n=98	n=99			
Baseline	19.6 (13.3, 25.9)	25.5 (19.5, 31.5)	21.6 (16.3, 27.0)	5.9 (-3.1, 14.9)	2.0 (-6.3, 10.4)	-3.9 (-12.0, 4.2)
4 months	15.2 (11.3, 19.2)	23.8 (19.3, 28.2)	25.0 (19.4, 30.6)	8.6 (2.4, 14.7)	9.8 (2.8, 16.8)	1.2 (-5.9, 8.4)
Change	-4.4 (-12.2, 3.5)	-1.7 (-7.3, 3.8)	3.4 (-5.5, 12.2)	2.6 (-7.0, 12.2)	7.7 (-4.0, 19.5)	5.1 (-5.3, 15.5)
**sTfR, mg/L**						
	n=70	n=99	n=99			
Baseline	4.17 (3.50, 4.83)	4.72 (4.25, 5.19)	4.51 (4.17, 4.84)	0.55 (-0.25, 1.36)	0.34 (-0.42, 1.10)	-0.21 (-0.81, 0.39)
4 months	4.91 (4.33, 5.49)	4.34 (4.13, 4.56)	4.47 (4.20, 4.75)	-0.57 (-1.21, 0.08)	-0.44 (-1.08, 0.21)	0.13 (-0.22, 0.48)
Change	0.74 (-0.24, 1.73)	-0.37 (-0.87, 0.12)	-0.03 (-0.53, 0.46)	-1.12 (-2.22, -0.02)	-0.78 (-1.88, 0.32)	0.34 (-0.36, 1.04)
**Hemoglobin, g/L**						
	n=73	n=105	n=104			
Baseline	110.5 (107.7, 113.3)	109.1 (105.8, 112.4)	110.2 (107.9, 112.6)	-1.4 (-5.8, 3.0)	-0.3 (-4.0, 3.5)	1.1 (-2.9, 5.2)
4 months	109.2 (107.3, 111.1)	113.0 (110.0, 116.1)	115.8 (114.2, 117.5)	3.8 (0.2, 7.4)	6.6 (3.9, 9.3)	2.8 (-0.8, 6.3)
Change	-1.3 (-4.1, 1.5)	3.9 (-1.7, 9.5)	5.6 (2.7, 8.4)	5.2 (-1.1, 11.5)	6.8 (2.8, 10.9)	1.6 (-4.7, 7.9)

95% confidence intervals are shown in parentheses. Estimates were obtained from multiple linear regressions using the natural log transformation for outcome variables, the linear predictor included an indicator variable of the 4 months period, indicator variables of study group (FF as reference category) and their interactions with study period. Study blocking indicator variables were included along with the other predictors. Standard errors were adjusted for data dependencies within communities. Geometric means, their changes and their comparisons were performed through predictive margins and their standard errors calculated with the Delta method. [1] MNP: Micronutrient powder.

Supplementary Table S3. Prevalence of zinc deficiency, low iron stores, tissue iron deficiency and anemia in baseline and follow up samples measured at 4 months without covariate adjustment, by study group.

	Fortified Food	Syrup	MNP[1]	Syrup vs FF	MNP vs FF	MNP vs Syrup
**Zinc deficiency (Zn < 9.9 µmol/L), %**						
	n=73	n=105	n=105			
Baseline	24.8 (15.0, 34.7)	41.0 (33.0, 48.9)	30.5 (23.1, 37.9)	16.1 (3.3, 28.9)	5.7 (-7.1, 18.4)	-10.4 (-21.8, 1.0)
4 months	11.9 (5.1, 18.8)	2.8 (0.2, 5.5)	8.0 (4.2, 11.8)	-9.1 (-16.5, -1.7)	-3.9 (-11.9, 4.1)	5.2 (0.6, 9.8)
Change	-12.9 (-24.3, -1.4)	-38.1 (-47.4, -28.8)	-22.5 (-32.7, -12.3)	-25.2 (-40.1, -10.3)	-9.6 (-25.0, 5.8)	15.6 (1.5, 29.8)
**Low iron stores (ferritin < 12 µg/L), %**						
	n=70	n=98	n=99			
Baseline	31.2 (18.9, 43.5)	28.9 (18.8, 39.1)	26.3 (19.1, 33.4)	-2.2 (-18.6, 14.1)	-4.9 (-19.3, 9.4)	-2.7 (-15.1, 9.7)
4 months	37.0 (25.0, 48.9)	15.9 (10.3, 21.5)	15.1 (7.0, 23.1)	-21.0 (-34.7, -7.4)	-21.9 (-36.5, -7.3)	-0.8 (-10.8, 9.1)
Change	5.8 (-11.0, 22.5)	-13.0 (-22.6, -3.4)	-11.2 (-24.5, 2.1)	-18.8 (-38.1, 0.5)	-17.0 (-38.4, 4.5)	1.8 (-14.6, 18.3)
**Tissue iron deficiency (sTfR > 6 mg/L), %**						
	n=70	n=99	n=99			
Baseline	15.1 (3.0, 27.2)	20.3 (10.0, 30.6)	18.3 (9.2, 27.3)	5.2 (-10.7, 21.1)	3.2 (-12.8, 19.2)	-2.0 (-16.1, 12.0)
4 months	15.1 (6.9, 23.3)	8.2 (2.7, 13.7)	6.6 (0.9, 12.3)	-6.9 (-16.8, 2.9)	-8.5 (-18.8, 1.7)	-1.6 (-9.6, 6.4)
Change	0.0 (-10.3, 10.3)	-12.1 (-25.1, 0.8)	-11.7 (-23.2, -0.2)	-12.1 (-28.6, 4.4)	-11.7 (-27.3, 3.9)	0.4 (-17.0, 17.8)
**Anemia (Hb < 110 g/L), %**						
	n=73	n=105	n=104			
Baseline	49.0 (42.4, 55.7)	48.7 (38.4, 59.0)	44.3 (33.8, 54.9)	-0.4 (-13.0, 12.3)	-4.7 (-18.1, 8.7)	-4.3 (-19.2, 10.5)
4 months	53.1 (46.4, 59.8)	37.2 (25.9, 48.5)	26.3 (20.6, 31.9)	-15.9 (-29.2, -2.7)	-26.9 (-36.1, -17.6)	-10.9 (-23.8, 1.9)
Change	4.1 (-4.1, 12.2)	-11.5 (-29.7, 6.7)	-18.1 (-30.0, -6.2)	-15.6 (-35.5, 4.4)	-22.2 (-36.6, -7.8)	-6.6 (-28.3, 15.0)

95% confidence intervals in parentheses. Estimates were obtained from logistic regressions, the linear predictor included an indicator variable of the 4 months period, indicator variables of study group (FF as reference category) and their interactions with study period. Study blocking indicator variables were included along with the other predictors. Standard errors in the model were adjusted for data dependencies within communities. Adjusted prevalence, their changes and their comparisons were performed through predictive margins and their standard errors calculated using the Delta method. [1] MNP: Micronutrient powder.
